# Short Communication: Dolutegravir-Based Regimens Are Active in Integrase Strand Transfer Inhibitor–Naive Patients with Nucleoside Reverse Transcriptase Inhibitor Resistance

**DOI:** 10.1089/aid.2017.0184

**Published:** 2018-04-01

**Authors:** James Demarest, Mark Underwood, Marty St. Clair, David Dorey, Dannae Brown, Andrew Zolopa

**Affiliations:** ^1^ ViiV Healthcare, Research Triangle Park, North Carolina.; ^2^ GlaxoSmithKline, Mississauga, Ontario, Canada.; ^3^ ViiV Healthcare, Abbotsford, Australia.

**Keywords:** HIV-1, dolutegravir, integrase inhibitor, raltegravir, antiretroviral resistance, combination antiretroviral therapy

## Abstract

In the SAILING study, dolutegravir demonstrated superior virologic efficacy compared with raltegravir in treatment-experienced, integrase strand transfer inhibitor (INSTI)–naive patients with HIV-1 who harbored resistance to ≥2 antiretroviral drug classes. Significantly fewer dolutegravir-treated patients demonstrated virologic failure with treatment-emergent resistance than raltegravir-treated patients through 48 weeks. Investigator-selected background therapy (ISBT) included at least one fully active agent, selected on the basis of resistance analysis. Genotypic and phenotypic resistance testing were performed on baseline and time-of-failure samples from patients with protocol-defined virologic failure (PDVF). A *post hoc* analysis of SAILING (*N* = 715; 354 dolutegravir, 361 raltegravir) assessed efficacy in subpopulations defined by ISBT activity, resistance profiles, and treatment history. When ISBT contained only nucleoside reverse transcriptase inhibitors (NRTIs), PDVF occurred in 0% (0/32) of dolutegravir-treated patients and 21.9% (7/32) of raltegravir-treated patients *(p* = .005*).* In patients harboring M184 V whose ISBT contained lamivudine or emtricitabine plus a second NRTI, 0% (0/13) of dolutegravir- and 33.3% (4/12) of raltegravir-treated patients *(p* = .026*)* experienced PDVF. Among patients receiving protease inhibitor (PI)–containing ISBT, 6.0% (18/300) of dolutegravir-treated patients versus 11.8% (36/305) of raltegravir-treated patients (*p* = .012) experienced PDVF. Darunavir/ritonavir was part of ISBT in 130 dolutegravir-treated patients and 145 raltegravir-treated patients; 6 (4.6%) and 12 (8.3%), respectively, experienced PDVF (difference −3.7%; 95% confidence interval: −10.1% to 2.5%; *p* = .256). There was no or less virologic failure in treatment-experienced, INSTI-naive subjects receiving dolutegravir versus raltegravir, even when the ISBT was suboptimal or NRTI resistance was present at baseline. These findings are not explained by the use of PI/ritonavir-containing ISBT.

Integrase strand transfer inhibitors (INSTIs) form a recent class of antiretroviral drugs approved for HIV-1 treatment.^1^ These agents (e.g., raltegravir, elvitegravir, dolutegravir) are included in combination antiretroviral therapy regimens recommended in the United States for initial treatment of HIV-1 infection.^2^ Each agent has demonstrated high virologic efficacy, favorable safety and tolerability profiles, and lack of cross-resistance to other antiretroviral classes; dolutegravir and raltegravir also exhibit a low incidence of drug–drug interactions.^1,2^

The phase III SAILING study (ClinicalTrials.gov identifier, NCT01231516) compared the clinical and virologic efficacy and safety of dolutegravir with raltegravir in treatment-experienced, INSTI-naive patients.^3^ Detailed procedures have been published. One criterion for inclusion in the SAILING study was evidence of resistance to ≥2 classes of antiretroviral drugs. Subjects' virologic profiles were used to create investigator-selected background therapy (ISBT) regimens to be used with dolutegravir or raltegravir. In the full-study analysis, there was a statistically significant lower rate of protocol-defined virologic failure (PDVF) with resistance among patients assigned to receive dolutegravir versus raltegravir (4 of 354 [1%] vs. 17 of 361 [5%]; adjusted difference, −3.7%; 95% confidence interval [CI]: −6.1 to −1.2; *p* = .003). We report the results of a *post hoc* analysis of the SAILING study cohort that examined virologic outcomes stratified by baseline resistance profiles, on-study components of ISBT administered with dolutegravir or raltegravir, and treatment history.

*Post hoc* statistical analyses were conducted using SAS, Version 9.4 (SAS Institute, Cary, NC). Exact tests (two-sided, Barnard's method) were used to compare proportions between treatment groups, and exact 95% CIs for the treatment difference (dolutegravir minus raltegravir) were calculated by the score method of Chan and Zhang,^4^ except for comparisons in subgroups defined by background regimen use of darunavir/ritonavir, for which CIs and *p* values were computed using a Wald normal approximation (for consistency with the methodology used in the primary article); *p* values were not adjusted for multiple comparisons.

Among patients receiving nucleoside reverse transcriptase inhibitor (NRTI)–only ISBT, no patients (0%, 0/32) in the dolutegravir group experienced PDVF compared with 22% (7/32) patients in the raltegravir group (*p* = .005; Fig. 1A). Rates of PDVF among patients with ISBT consisting of two fully active NRTIs were 0% (0/16) in the dolutegravir group and 15.8% (3/19) in the raltegravir group (difference −15.8%; 95% CI: −39.6 to 5.9; *p* = .115). For patients with NRTI-only ISBT contained one fully active NRTI, PDVF occurred in 0% of patients (0/12) treated with dolutegravir and 30.8% of patients (4/13) treated with raltegravir (*p* = .045).

**FIG. 1.**
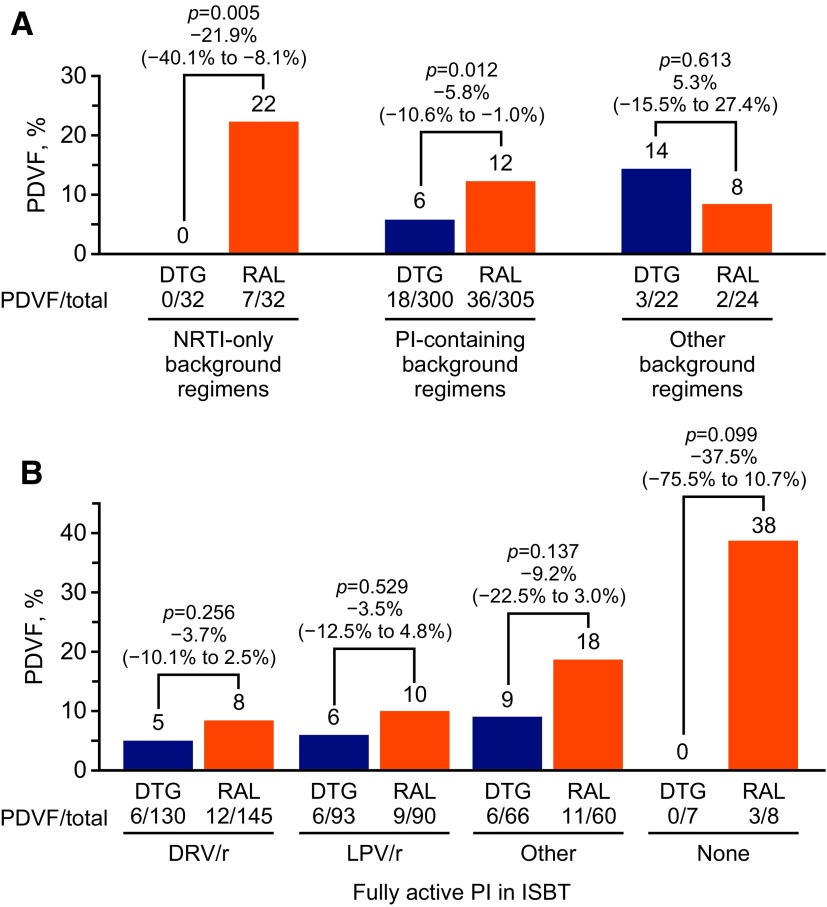
**(A)** Cumulative incidence of PDVF at week 48 by type of ISBT. **(B)** Cumulative incidence of PDVF in patients receiving ISBT containing a PI. Labels above individual bars denote percentage of patients who experienced PDVF, and labels above pairs of bars show the *p* value, treatment difference, and 95% confidence interval. In addition to the patients represented here, four patients in the dolutegravir group (0 PDVF) and two patients in the raltegravir group (1 PDVF) had missing phenotypes. DRV/r, darunavir/ritonavir; DTG, dolutegravir; ISBT, investigator-selected background therapy; LPV/r, lopinavir/ritonavir; NRTI, nucleoside reverse transcriptase inhibitor; PDVF, protocol-defined virologic failure; PI, protease inhibitor; RAL, raltegravir.

Among patients infected with HIV-1 variants with thymidine analog mutations (TAMs), PDVF occurred in 6.1% (10/164) of patients in the dolutegravir group and 10.2% (17/166) of patients in the raltegravir group (difference −4.1%; 95% CI: −10.5 to 2.0; *p* = .211). Of the subpopulation of patients with TAMs who received ISBT that included a less-than-fully active NRTI as a second agent, PDVF occurred in 12.5% (3/24) of patients treated with dolutegravir and 5.3% (1/19) of patients treated with raltegravir (difference 7.2%; 95% CI: −14.8 to 27.8; *p* = .588). Among patients with the M184V mutation detected at baseline who received an NRTI-only ISBT containing either lamivudine or emtricitabine along with a second NRTI (dolutegravir group: abacavir, *n* = 2; tenofovir, *n* = 8; zidovudine, *n* = 3; raltegravir group: abacavir, *n* = 6; tenofovir, *n* = 4; zidovudine, *n* = 2), 0% (0/13) of patients treated with dolutegravir and 33.3% (4/12) of patients treated with raltegravir experienced PDVF (*p* = .026); the second NRTI used in these 12 patients was either abacavir (*n* = 6), tenofovir (*n* = 4), or zidovudine (*n* = 2), and the 4 raltegravir PDVFs were spread across these NRTIs. In addition, 3 of the 13 dolutegravir-treated patients also had ≥2 TAMs and PDVF did not occur in any of these patients. For the 12 patients treated with raltegravir, 1 patient had 1 TAM and 1 patient had ≥2 TAMs; PDVF occurred in the patient with 1 TAM.

A boosted protease inhibitor (PI) was included in the ISBT of 300 patients (84.7%) in the dolutegravir group and 305 patients (84.5%) in the raltegravir group, of which 18 (6.0%) and 36 (11.8%) experienced PDVF (*p* = .012), respectively (Fig. 1B). Most of these patients (289 in the dolutegravir group and 295 in the raltegravir group) were on background regimens that included one fully active PI, and PDVF occurred in 18 patients (6.2%) treated with dolutegravir and 32 patients (10.8%) treated with raltegravir (*p* = .047). Rates of PDVF among patients with fully active darunavir/ritonavir in their ISBT were 4.6% (6/130) in the dolutegravir group and 8.3% (12/145) in the raltegravir group (difference –3.7%; 95% CI: −10.1 to 2.5; *p* = .256; Fig. 1B). For patients who received lopinavir/ritonavir, PDVF occurred in six patients (6.5%) treated with dolutegravir and nine patients (10.0%) treated with raltegravir (difference –3.5%; 95% CI: −12.5 to 4.8; *p* = .529). For other PIs, PDVF rates were 9.1% (6/66) for dolutegravir and 18.3% (11/60) for raltegravir (difference –9.2%; 95% CI: −22.5 to 3.0; *p* = .137). When no fully active PI was included in the background regimen, no PDVFs occurred in the seven patients treated with dolutegravir, whereas 37.5% (3/8) in the raltegravir group experienced PDVF (difference –37.5%; 95% CI: −75.5 to 10.7; *p* = .099).

Efficacy was also examined by comparing virologic response rates in the dolutegravir and raltegravir groups when the background regimen did or did not include darunavir/ritonavir. Most patients did not receive darunavir/ritonavir-containing ISBT (*N* = 423; 214 dolutegravir, 209 raltegravir), and the response rates for these subsets were 66.8% (*n* = 143) for dolutegravir and 60.3% (*n* = 126) for raltegravir (difference 6.5%; 95% CI: −2.6 to 15.7; *p* = .162; based on normal approximation). As previously reported in the primary article,^3^ the proportion of virologic responders among patients infected with PI-resistant virus treated with darunavir/ritonavir was 85.3% (58/68) in the dolutegravir group and 66.7% (50/75) in the raltegravir group, resulting in a statistically significant treatment difference of 18.6% (95% CI: 5.0%–32.2%; *p* = .007). The virologic response rates were similar among patients treated with darunavir/ritonavir who were not infected with PI-resistant HIV-1 (dolutegravir, 50/72 [69.4%]; raltegravir, 54/77 [70.1%]; difference –0.7%; 95% CI: −15.4 to 14.1; *p* = .927). Although further study is needed, the larger numerical difference in the virologic response rate between dolutegravir and raltegravir among subjects for whom darunavir was not fully active, in contrast to the corresponding comparisons among subjects with fully active darunavir, suggests that fully active darunavir may mask differences in virologic activity between dolutegravir and raltegravir.

When initiating HIV-1 treatment, patients and healthcare providers should consider multiple factors that determine how well a regimen suits the patient's individual needs. These choices impose a considerable burden on patients because factors related to adherence, treatment effectiveness, and barrier to resistance can influence the odds of treatment failure and its associated health consequences. Dolutegravir has demonstrated a high barrier to resistance in treatment-naive patients,^5–9^ and the SAILING study has demonstrated similar findings in treatment-experienced, INSTI-naive patients.^3^ The *post hoc* analyses reported here highlight the high virologic efficacy of dolutegravir, even when the choice of ISBT is complicated by virologic resistance, treatment history, or the inclusion of background agents with suboptimal activity. Although this was a *post hoc* analysis of small subgroups, the data showed that in the majority of comparisons, patients treated with dolutegravir had either no PDVFs or fewer compared with raltegravir-treated patients. The lower frequency of PDVFs in subjects receiving dolutegravir could suggest a potency difference, a higher barrier to resistance, or a difference in adherence; however, we are not able to determine which/if any factors had a role in the patterns reported here.

Several key findings highlighted in this analysis warrant continued investigation. First, no patients in the dolutegravir group experienced PDVF while on ISBT that included only NRTIs. Second, no patients affected by NRTI resistance as a consequence of TAMs or the M184V variant of HIV-1 experienced PDVF while being treated with dolutegravir with an ISBT that only contained NRTIs. Third, observed dolutegravir efficacy was not explained by the use of ISBT containing darunavir/ritonavir, lopinavir/ritonavir, or other boosted PI, even among patients infected with HIV-1 with primary PI resistance mutations.

Our findings support the clinical utility of dolutegravir with several classes of background agents and suggest that dolutegravir-based regimens can reduce virological failure, even in patients with complicated virologic profiles. Although these findings are likely to reflect clinical situations in which dolutegravir may be a viable option to recover or maintain virologic suppression, additional data are needed to understand the efficacy of dolutegravir-based regimens in settings in which the ISBT is not fully active.
